# Overall and Cause‐Specific Mortality Among Patients With Cutaneous T‐Cell Lymphoma in the United States

**DOI:** 10.1002/jha2.1099

**Published:** 2025-03-04

**Authors:** Lauren Shea, Mayur Narkhede, Karthik Chamarti, Tina Gao, Amitkumar Mehta, Gaurav Goyal

**Affiliations:** ^1^ Division of Hematology and Oncology University of Alabama at Birmingham Birmingham Alabama USA; ^2^ Division of Hematology and Oncology The University of Texas Health Science Center at San Antonio San Antonio Texas USA; ^3^ Vestavia High School Vestavia Hills Alabama USA

**Keywords:** cause of death, mycosis fungoides, primary cutaneous anaplastic large cell lymphoma, Sézary syndrome, subcutaneous panniculitis‐like T‐cell lymphoma

## Abstract

**Introduction:**

Little is known about long‐term outcomes and causes of death for individuals with cutaneous T‐cell lymphoma.

**Methods:**

We used SEER‐18 registry data to examine outcomes among 9886 adults with mycosis fungoides (MF), Sézary syndrome (SS), primary cutaneous anaplastic large cell lymphoma (pcALCL), and subcutaneous panniculitis‐like T‐cell lymphoma (SPTCL) diagnosed from 2000 to 2018. We calculated overall survival (Kaplan–Meier method), standardized mortality ratios (SMRs), absolute excess risk (AER) of death, and cumulative incidence of cause‐specific mortality.

**Results:**

Individuals with CTCL were at increased risk of all‐cause mortality relative to age‐matched controls, with SMR ranging from 1.57 (95% CI: 1.49–1.65) in MF to 5.61 (95% CI: 4.65–6.7) in SS. This was true even for those who initially presented with early‐stage disease. After a median follow‐up of 64 months, the cumulative incidence of lymphoma‐related death was 16.5%, compared to 10.5% other causes, 9.6% cardiovascular, 9.1% second primary malignancy, 1.8% infection, and 1.1% unknown cause. People diagnosed with CTCL were at higher risk of mortality due to leukemia and infectious causes than control populations, but secondary causes made overall minor contributions to total mortality.

**Conclusion:**

This population‐level analysis revealed that individuals with CTCL were at increased risk of all‐cause mortality relative to age‐matched controls and that lymphoma remained a significant cause of death even in those presenting with early‐stage disease.

**Trial Registration:**

The authors have confirmed clinical trial registration is not needed for this submission.

## 1 Introduction

Cutaneous T‐cell lymphomas (CTCL) are rare subtypes of non‐Hodgkin lymphoma characterized by primary cutaneous disease, though the involvement of blood, lymph nodes, and visceral organs can occur in advanced stages. The most common subtype is mycosis fungoides (MF). Sézary syndrome (SS) is a leukemic variant characterized by peripheral blood involvement with malignant lymphocytes with a similar immunophenotype [1, 2]. Less common subtypes of CTCL include primary cutaneous anaplastic large cell lymphoma (pcALCL) and subcutaneous panniculitis‐like T‐cell lymphoma (SPTCL). The clinical course of CTCL is highly heterogeneous. Though many patients have relatively indolent diseases that can be effectively managed with skin‐directed therapy for years, others suffer from an aggressive course similar to that seen in peripheral T‐cell lymphoma. Prior studies have employed single‐center retrospective registries [3, 4, 5, 6, 7, 8, 9], multicenter registries [10, 11, 12, 13, 14, 15], or population‐level analyses [2, 16–21] to examine predictors of overall survival (OS) in CTCL. A prior analysis of data from the Survival, Epidemiology, and End‐Results (SEER) registry revealed that among patients with MF, non‐Hodgkin lymphoma was the most common cause of death regardless of stage at presentation [21]. However, there is a lack of data on cause‐specific mortality in rarer subtypes of CTCL at the population level, which represents a critical knowledge gap for the care of these individuals. In this study, we used the SEER‐18 dataset to investigate overall and cause‐specific survival in a large cohort of individuals with CTCL from the United States.

## 2 Methods

### 2.1 Data Source and Cohort Selection

The cohort was derived from the November 2018 submission of the SEER registry. Cases were selected based on the International Classification of Diseases for Oncology, third edition (ICD‐O‐3) codes as follows: MF(9700), SS (9701), pcALCL (9718), and SPTCL (9708). Patients were included if they were diagnosed between 2000 and 2018 and were 15 years or older at the time of diagnosis. Patients with unknown age at diagnosis (*n* = 0) and/or unknown race (*n* = 493) were excluded. The SEER*Stat Rate Session and Case Listing Session were used to collect baseline characteristics of the cohort.

The variable “Derived AJCC Stage Group, sixth edition” was used to classify patients into early stage (IA, IB, and IIA) or advanced stage (IIB or greater).

### 2.2 Overall and Cause‐Specific Mortality Analysis

OS was defined as the time from diagnosis to death or censoring at the last follow‐up. The SEER*Stat Survival Session was used to determine 2‐ and 5‐year OS and median OS. The SEER*Stat Multiple Primary‐SIR Session was used to determine the standardized mortality ratio (SMR) and absolute increased risk (AER) for death. SMR was defined as the ratio of the observed number of deaths in the study cohort attributed to a given cause to the expected number of deaths in the control population attributed to the same cause. The AER was defined as the number of excess deaths attributed to a certain cause in the study cohort beyond the expected number of deaths attributed to the same cause in the control population, per 1000 persons per year, or [(observed deaths − expected deaths) × 1000]/person years at risk. The incidence of cause‐specific mortality was estimated using cumulative incidence functions with Fine–Gray model (R Statistical Software, Version 4.3.1). Survival curves for individuals with different subtypes of CTCL and for Black and White individuals with a given subtype of CTCL were compared using log rank test (R Statistical Software, Version 4.3.1).

## 3 Results

### 3.1 Incidence and OS

The study cohort was comprised of 9886 individuals with CTCL (80.5% MF, 2.8% SS, 14.7% pcALCL, and 2.1% SPTCL). Median follow‐up in months was 66 (range: 0–227) for MF, 26 (range: 0–216) for SS, 66 (range: 0–226) for pcALCL, and 48 (range: 0–218) for SPTCL (Table 1). Age‐adjusted incidence (per 1,000,000) was 6.1 for MF, 0.21 for SS, 1.1 for pcALCL, and 0.16 for SPTCL. The incidence of MF, SS, and SPTCL appeared to be higher in Black individuals compared with other races (Table S1). Five‐year OS was 83.6% for MF, 79.9% for cALCL, and 71.3% for SPTCL. Individuals with SS had the lowest 5‐year OS (38.4%; Table 1). Black race was associated with inferior OS relative to White in MF, SS, and pcALCL, though not in SPTCL (Table S2). Survival outcomes were similar among males and females in each subtype (data not shown).

**TABLE 1:** Incidence and Kaplan–Meier analysis by CTCL subtype.

	MF	SS	pcALCL	SPTCL
Patients diagnosed	7957	272	1452	205
Incidence (per 1,000,000)	6.1	0.21	1.1	0.16
Patient deaths	1474	120	470	69
Follow‐up in months (median, range)	66, 0–227	26, 0–216	66, 0–226	48, 0–218
2‐year OS, %	93.0	67.7	88.0	79.0
5‐year OS, %	83.6	38.4	79.9	71.3
Median OS in months	NR	44.1	NR	NR

Abbreviations: MF, mycosis fungoides; NR, not reached; OS, overall survival; pcALCL, primary cutaneous anaplastic large cell lymphoma; SPTCL, subcutaneous panniculitis‐like T‐cell lymphoma; SS, Sézary syndrome.

### 3.2 Causes of Death

**FIGURE 1:**
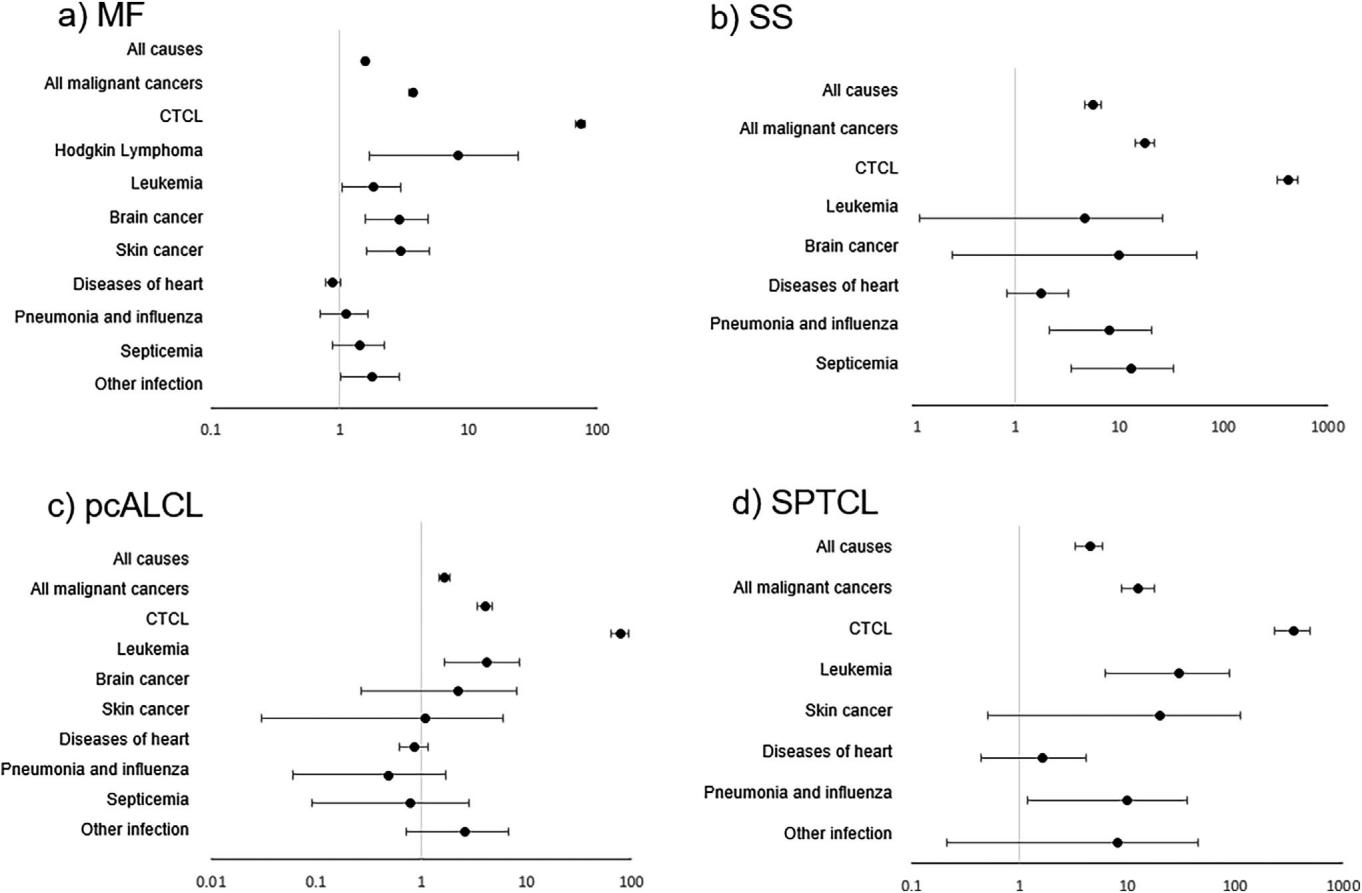
Standardized mortality ratios (SMR) by CTCL subtype. CTCL, cutaneous T‐cell lymphoma; MF, mycosis fungoides; pcALCL, primary cutaneous anaplastic large cell lymphoma; SPTCL, subcutaneous panniculitis‐like T‐cell lymphoma; SS, Sézary syndrome.

**FIGURE 2:**
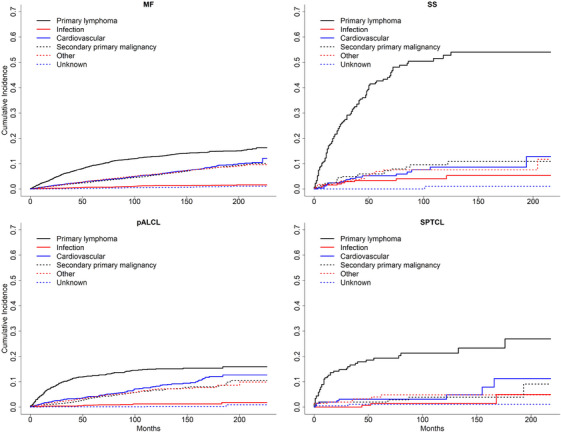
Cumulative incidence of cause‐specific mortality by CTCL subtype. CTCL, cutaneous T‐cell lymphoma; MF, mycosis fungoides; pcALCL, primary cutaneous anaplastic large cell lymphoma; SPTCL, subcutaneous panniculitis‐like T‐cell lymphoma; SS, Sézary syndrome.

Individuals with CTCL were at an increased risk of all‐cause mortality relative to age‐matched controls in all subtypes of CTCL examined (Figure 1; Table S4). SMR was 1.57 (95% CI: 1.49–1.65) for MF, 5.61 (4.65–6.7) for SS, 1.66 (1.48–1.86) for pcALCL, and 4.45 (3.26–5.93) for SPTCL. CTCL accounted for the greatest proportion of deaths in all CTCL subtypes examined, with death attributed to CTCL in 40.5% (MF), 66.7% (SS), 40.0% (pcALCL), and 65.2% (SPTCL) of reported deaths (Figure 2; Table S3). Individuals with CTCL were also at increased risk of death from select second primary cancers. The risk of mortality from leukemia was increased in MF (SMR 1.80; 95% CI: 1.03–2.93), pcALCL (SMR 4.16; 1.67–8.57), and SPTCL (SMR 29.74; 6.13–86.91) but not in SS (SMR 4.7; 0.12–26.16). Individuals with MF were also at increased risk of death from skin cancer (SMR 2.96; 1.62–4.97), brain cancer (SMR 2.87; 1.57–4.82), and Hodgkin lymphoma (8.20, 1.69–23.96), but this was not seen in the other subtypes of CTCL examined (Figure 1). The risk of mortality from select infectious causes was also increased in several subtypes of CTCL, including septicemia in SS (SMR 12.77; 95% CI: 3.48–32.69), pneumonia and influenza in SS (SMR 7.87; 2.14–20.14), “other” infections in MF (SMR 1.77; 1.01–2.87), and pneumonia and influenza in SPTCL (SMR 9.80; 1.19–35.42). Though individuals with CTCL were at increased risk of death from several types of secondary malignancies relative to age‐matched controls, these secondary malignancies made relatively minor contributions to the burden of mortality. For example, in MF, the AER of death due to primary lymphoma was 13.2 per 1000 per year, as compared to 0.159 from leukemia (Figure 2; Table S4). Infectious causes similarly made a relatively minor contribution to overall mortality as compared to the primary CTCL (Figure 2), with AER < 5 (MF, ALCL, and SPTCL) and < 10 (SS; Table S4).

### 3.3 Causes of Death by Race

Among individuals with MF, all racial groups had a higher risk of all‐cause mortality than age‐matched controls, but this was most dramatic for Black individuals, with SMR 2.46 (95% CI: 2.17–2.77) for Blacks versus 1.44 (95% CI: 1.36–1.53) for Whites (Table S5). The same pattern was observed for SS and pcALCL but not for SPTCL. Though the overall cohort of MF did not have an increased risk of death due to septicemia, the subset of Black individuals did, with SMR 2.97 (95% CI: 1.19–6.12; Table S5). However, this was not the case in the other subsets of CTCL examined. In SS, Whites were at increased risk of mortality from septicemia (SMR 14.39; 95% CI: 3.92–36.85), whereas Blacks were not (SMR 0.00; 95% CI: 0–109.74).

### 3.4 Causes of Death by Latency

In all examined subtypes of CTCL, the most common causes of death shifted over time from initial diagnosis. People who died within the first year of diagnosis were more likely to have death attributed to CTCL, whereas non‐lymphoma causes were more common later on. For example, in MF, in the first 2–11 months after diagnosis, 61.4% of deaths were due to CTCL, compared to 48.5%, 30.7%, and 17.7% of deaths that occurred 12–59, 60–119, and > 120 months from diagnosis, respectively (Table S6). A similar trend was seen in SS, pcALCL, and SPTCL (Table S6).

### 3.5 Cause of Death by Age

We examined causes of death by histology based on age at diagnosis, age 15–64 (younger) versus age ≥ 65 (older). For people with both SS and SPTCL, the majority of deaths were attributable to CTCL in both the younger and older groups. In MF and pcALCL, in contrast, whereas CTCL accounted for the majority of deaths in the younger group, noncancer causes surpassed CTCL as a cause of death in the older group (Figure S1).

### 3.6 Causes of Death by Stage

**FIGURE 3:**
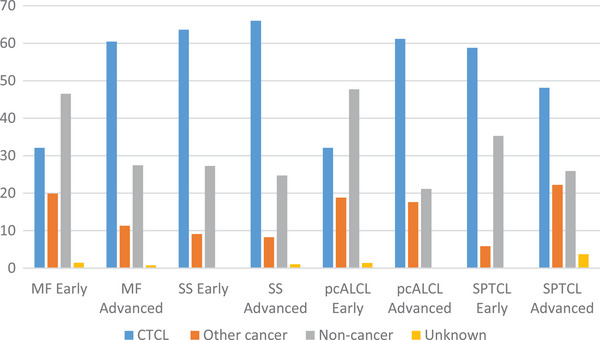
Causes of death by stage and CTCL subtype. Graph presents the percentage of deaths, per CTCL subtype and stage, attributed to the primary lymphoma, to second primary cancers, and to noncancer causes. CTCL, cutaneous T‐cell lymphoma; MF, mycosis fungoides; pcALCL, primary cutaneous anaplastic large cell lymphoma; SPTCL, subcutaneous panniculitis‐like T‐cell lymphoma; SS, Sézary syndrome.

Across all subtypes of CTCL in our cohort, the stage at diagnosis was early (IA, IB, or IIA) in 34.3%, advanced (Stage IIB or greater) in 8.5%, and unknown in 57.1%. In all subtypes of CTCL, the risk of all‐cause mortality was increased relative to age‐matched controls, even for those who initially presented with early‐stage disease. Among those with early stage at presentation, SMR was 1.2 (95% CI: 1.1–1.3) in MF, 1.3 (1.1–1.5) in pcALCL, and 3.1 (1.6–5.4) in SPTCL (Table S7). The number of individuals with SS classified as early stage was too small to draw meaningful conclusions. As expected, the risk of death from CTCL was higher for those who initially presented with advanced stage as opposed to early‐stage disease. 60.4% of deaths in individuals with MF who presented with advanced stage disease were attributed to CTCL, compared to 32.1% of deaths in those who presented with early‐stage disease (Figure 3; Table S7). A similar percentage of deaths in individuals with initially early‐stage pcALCL was attributed to CTCL (32.1%). For individuals with SPTCL, the percentage of deaths attributed to CTCL was similar regardless of initial presentation with early versus advanced stage (Figure 3; Table S7).

## 4 Discussion

This analysis of more than 9000 individuals with CTCL revealed an increased risk of all‐cause mortality relative to age‐matched population controls. We also provide the first population‐level analysis of cause‐specific mortality in SS, pcALCL, and SPTCL, showing that though individuals with CTCL are at increased risk of mortality from select second primary cancers and from infectious causes, the cumulative mortality attributed to the primary lymphoma exceeds that from all other causes.

When comparing individuals who initially presented with early versus advanced‐stage MF and pcALCL, a higher proportion of deaths among those initially presenting with advanced‐stage disease was attributed to lymphoma. Our data are consistent with that reported from a single‐institution retrospective study of 525 patients with MF/SS, wherein the relative risk for death increased with increasing stage [3]. In this study, only 2% of deaths of those initially presented with Stage 1A disease were attributed to lymphoma, as compared to 90% of deaths among those with Stage IV extracutaneous disease. In contrast, a prior SEER analysis of patients with MF diagnosed between 2000 and 2016 showed that lymphoma was the most common cause of death across all stages, with 33% of deaths among those with Stage IA disease attributed to lymphoma versus 73% of those with Stage IV disease [21]. In our cohort, a substantial proportion of deaths in those presenting with early‐stage disease was attributed to lymphoma (32.1% in MF, 32.1% in pcALCL, and 58.8% in SPTCL). Somewhat surprisingly, even patients with early‐stage disease at presentation had an increased risk of all‐cause mortality relative to the general population. We also found that most of the deaths within the first year of CTCL diagnosis were attributed to lymphoma compared with other causes (Table S6). This information is important for clinicians to counsel newly diagnosed patients accurately, and it underscores the importance of appropriate follow‐up and monitoring regardless of the stage of diagnosis.

Regarding second primary cancers, individuals with three subtypes of CTCL included in this study (MF, pcALCL, and SPTCL) were at an increased risk of mortality due to leukemia relative to age‐matched controls, though leukemia made a relatively small contribution to total excess risk of mortality compared to primary lymphoma. A previous study from the SEER registry also highlighted a high risk of second cancers among patients with diverse T‐cell neoplasms, including CTCL [22]. Our data indicated that patients with MF were at increased risk of death due to skin cancer relative to controls. However, the deaths due to skin cancer were attributed to “other” skin cancers, excluding basal cell, squamous cell, and melanoma, so we cannot exclude the possibility that these were actual deaths due to CTCL. Notably, people with CTCL were not at increased risk of death from cardiovascular causes relative to age‐matched controls, in contrast to what has been observed in Hodgkin lymphoma and systemic non‐Hodgkin lymphoma [23, 24]. This may be due to the fact that patients with CTCL are rarely exposed to mediastinal radiation, which has been associated with an increased risk of late mortality from cardiovascular causes in other lymphoma subtypes [23, 24]. Patients with CTCL are frequently treated with retinoids, for which hypercholesterolemia is a known adverse effect. Though some studies have detected an increased risk of myocardial infarction and stroke in patients with CTCL [25], other studies have not confirmed this association [9]. Though we did not observe an increased risk of death due to cardiovascular causes in this cohort, it is possible that our follow‐up (median 26–66 months, depending on histology) was too short to detect the emergence of delayed cardiovascular toxicities.

In this cohort, infectious causes made a relatively small contribution to total mortality, with 4.1% of total deaths due to infectious causes as compared to 42.6% due to lymphoma. This is in contrast to smaller single‐institution studies were the percentage of total deaths due to infection ranged from 27% to 58% [8, 26–28]. There are several possible explanations for these findings. Changes in treatment practices, including a shift away from conventional chemotherapy and toward targeted therapies, may account for the decreased frequency of infectious deaths that we observed. As we cannot review details of individual cases in our cohort, we cannot exclude the possibility that some of the deaths attributed to lymphoma also had infection as a contributing cause.

Unsurprisingly, the SS cohort in our study had the poorest OS of the subtypes of CTCL examined (5‐year OS, 38.4%). CTCL was by far the most prevalent cause of death in individuals with SS, with 66.7% of deaths attributed to the primary lymphoma. These outcomes were inferior to those reported in a multicenter retrospective series, including 178 patients with SS diagnosed between 2012 and 2020, where the 5‐year OS was 53.4% [15]. The superior survival in the cohort described by Campbell et al. [15] (derived from three quaternary referral centers in Europe) relative to our cohort (derived from various practice settings in the United States) may be due in part to differential access to therapies such as clinical trials and allogeneic stem cell transplantation. As detailed treatment data are not available for our cohort, we are unable to test this hypothesis using this dataset.

Our data suggest that despite an overall favorable prognosis, a significant proportion of patients with pcALCL ultimately suffer from lymphoma‐related mortality. Our findings complement prior registry and single‐institution studies. A SEER analysis of patients with pcALCL diagnosed between 2005 and 2016 revealed a 5‐year OS of 80.6% [17], similar to the 79.9% 5‐year OS in the current study. In a retrospective analysis of 47 patients with pcALCL from the British Columbia Cancer Agency Lymphoid Cancer Database, the 5‐year OS was 75%, with 4 of 14 deaths (29%) due to pcALCL [16]. A single‐institution study of 48 patients with pcALCL showed a 5‐year OS of 76%, with 5 of the 10 deaths during the follow‐up period due to pcALCL [4]. In contrast, a multicenter retrospective analysis of 95 individuals with pcALCL treated at eight academic medical centers revealed a 5‐year OS of 77%, with only 3% of deaths attributed to lymphoma [14]. In summary, though these analyses consistently show 5‐year OS of approximately 75%–80% in pcALCL, the percentage of deaths due to primary lymphoma varies significantly. We cannot exclude the possibility that some of the deaths attributed to pcACLC in our SEER cohort were incorrectly assigned or that patients with systemic ALCL with skin involvement were erroneously classified as pcALCL in the SEER registry. However, the relatively high percentage of deaths attributed to CTCL in our cohort is more in keeping with the majority of retrospective analyses performed to date [4, 16, 17]. The high pcALCL‐related cumulative mortality in the current study suggests the need for better therapies for this rare subtype of T‐cell lymphoma.

Our study revealed a 5‐year OS of 71.3% for patients with SPTCL, with 65.2% of deaths attributed to the primary malignancy. SPTCL (characterized by an alpha–beta T‐cell phenotype) has only recently been distinguished from the immunophenotypically and clinically distinct entity primary cutaneous gamma–delta T‐cell lymphoma (GDTCL), which is associated with an inferior prognosis [1, 11]. The presence of associated hemophagocytic lymphohistiocytosis (HLH) is prognostic in SPTCL, with a 5‐year OS of 46% for those presenting with HLH versus 91% for those without HLH in one early retrospective series [11]. Despite overall favorable outcomes, the majority of deaths of patients with SPTCL were attributed to lymphoma in this study. In a more recent multi‐institution retrospective study of patients with SPTCL (*n* = 95), no patients had died due to disease progression or HLH after a median follow‐up of 56 months [29]. An analysis of patients with SPTCL included in the National Cancer Database (*n* = 353) likewise showed excellent outcomes overall with median OS > 10 years; causes of death were not specified in this analysis [18]. We cannot exclude the possibility that a subset of the SPTCL cohort in the current study might actually be classified as GDTCL by current WHO criteria, thereby contributing to the relatively high incidence of lymphoma‐specific mortality (21.3% at 100 months) that we observed. However, it is also possible that the differences in outcomes observed in our SEER cohort and the cohort reported by Guitart et al. [29] (derived from several major academic medical centers) could be due to demographic, socioeconomic, and/or treatment differences between the two cohorts.

Our study has several limitations. The SEER registry does not capture granular data on treatments received, and we are therefore unable to determine if the increased risk of particular second primary cancers is associated with different treatment modalities. These data do not capture precise TNM staging for CTCL (e.g., blood involvement is not captured), and more than 50% of our cohort had an unknown TNM stage at diagnosis. In addition, SEER data do not describe the evolution/progression from one stage to another over a patient's lifetime. Therefore, whether patients with initially localized disease who experienced lymphoma‐related mortality had progressed to a more advanced stage at the time of death cannot be determined from this dataset. The strengths of our study include the large number of individuals with rare subtypes of NHL, which we were able to capture in our analysis. In addition, our data are derived from a population‐based registry, which provides a global view when outcomes could theoretically differ based on whether patients receive care at community practices versus large academic centers. In the future, well‐conducted prospective registries capturing a diverse cohort of CTCL patients will be essential to answer key outstanding questions, such as the association between certain treatment modalities and secondary malignancies. The Prospective Cutaneous Lymphoma International (PROCLIPI) study, launched in 2015, is one such effort that has already yielded important insights into CTCL treatment and prognosis [30, 31].

In conclusion, our analysis of SEER data revealed that individuals with CTCL were at increased risk of all‐cause mortality relative to age‐matched controls. This was true even for those presenting with early‐stage disease. Despite an overall favorable prognosis in MF, pcALCL, and SPTCL, lymphoma‐related deaths accounted for the majority of the excess risk of mortality. Though individuals with CTCL were at higher risk of mortality due to leukemia (MF, pcALCL, and SPTCL) and various infectious causes (MF, SS, and SPTCL) than control populations, these secondary causes made overall minor contributions to total mortality. Our data support the need for improved therapies and close long‐term follow‐up of patients with CTCL regardless of stage at presentation. Future studies should aim to evaluate interventions to reduce overall mortality.

## Author Contributions

L.S. performed the research. K.C. and M.N. provided assistance with statistical analysis. T.G. generated Figure 2. L.S. and G.G. analyzed the data and wrote the paper. M.N. and A.M. provided essential feedback during manuscript preparation.

## Ethics Statement

This study was conducted with IRB approval from the University of Alabama at Birmingham.

## Consent

The authors have nothing to report.

## Conflicts of Interest

The authors declare no conflicts of interest.

## Supporting information



Supporting Information

## Data Availability

Data are fully available upon request to the corresponding author.
