# Supplementary material for: Overall and Cause‐Specific Mortality Among Patients With Cutaneous T‐Cell Lymphoma in the United States
Source: EJHaem. 2025 Mar 4;6(2):e1099. doi: 10.1002/jha2.1099 (PMC11876778; doi:10.1002/jha2.1099)
Supplement: Supplementary file 1 — Supporting Information [file JHA2-6-e1099-s001.docx]

**Supplemental Figures**

**Figure S1.** Causes of death by age at diagnosis and CTCL subtype

Graph presents the percentage of deaths, per CTCL subtype and age, attributed to the primary lymphoma, to second primary cancers, and to non-cancer causes.

CTCL: Cutaneous T-cell lymphoma; MF: Mycosis fungoides; pcALCL: Primary cutaneous anaplastic large cell lymphoma; SPTCL: Subcutaneous panniculitis-like T-cell lymphoma; SS: Sézary syndrome.

**Supplemental Tables**

|  | MF | SS | pcALCL | SPTCL |
| --- | --- | --- | --- | --- |
| All | 6.1 | 0.21 | 1.1 | 0.16 |
| White | 5.9 | 0.21 | 1.2 | 0.12 |
| Black | 9.0 | 0.36 | 1.2 | 0.32 |
| AI AN | 2.8 | 0.28 | 0.60 | 0.16 |
| Asian | 4.4 | 0.06 | 0.58 | 0.23 |

**Table S1.** Age-adjusted incidence (per 1,000,000) by CTCL subtype and ethnicity.

AI AN: American Indian / Alaska Native; CTCL: Cutaneous T-cell lymphoma; MF: Mycosis fungoides; pcALCL: Primary cutaneous anaplastic large cell lymphoma; SPTCL: Subcutaneous panniculitis-like T-cell lymphoma; SS: Sézary syndrome.

|  | Number (%) | 2 year | 5 year | Median (mo) | KM analysis comparing OS for White versus Black individuals (log-rank *p*) |
| --- | --- | --- | --- | --- | --- |
| MF | | | | | |
| All | 6,499 (100) | 93.0 | 83.6 | NR |  |
| White | 4,862 (74.8) | 93.3 | 83.7 | NR |  |
| Black | 1,100 (16.9) | 90.5 | 79.8 | NR | 0.04 |
| AI AN | 36 (0.6) | 91.1 | 79.8 | NR |  |
| Asian | 501 (7.8) | 96.1 | 91.0 | NR |  |
| SS | | | | | |
|  | Number (%) | 2 year | 5 year | Median (mo) |  |
| All | 203 (100) | 67.6 | 38.4 | 44.1 |  |
| White | 159 (78.3) | 70.7 | 41.4 | 48.2 |  |
| Black | 36 (17.7) | 55.8 | 23.6 | 26.7 | 0.02 |
| AI AN | 2 (1.0) | 1 | 0.5 | 36 |  |
| Asian | 6 (3.0) | 0.4 | 0.4 | 21.5 |  |
| pcALCL |  | | | | |
|  | Number (%) | 2 year | 5 year | Median (mo) |  |
| All | 1155 (100) | 88.0 | 79.9 | NR |  |
| White | 955 (82.7) | 88.3 | 80.6 | NR |  |
| Black | 128 (11.1) | 80.8 | 68.8 | NR | 0.02 |
| AI AN | 7 (0.6) | 1 | 83.3 | NR |  |
| Asian | 65 (5.6) | 96.7 | 90.2 | NR |  |
| SPTCL |  | | | | |
|  | Number (%) | 2 year | 5 year | Median (mo) |  |
| All | 178 (100) | 79.0 | 71.3 | NR |  |
| White | 108 (60.7) | 74.3 | 61.9 | 168.0 |  |
| Black | 37 (20.8) | 77.3 | 77.3 | NR | 0.5 |
| AI AN | 3 (1.7) | 100 | 100 | NR |  |
| Asian | 30 (16.9) | 96.3 | 96.3 | NR |  |

**Table S2.** Kaplan-Meier analysis by CTCL subtype and ethnicity

AI AN: American Indian / Alaska Native; CTCL: Cutaneous T-cell lymphoma; MF: Mycosis fungoides; NR: Not reached; pcALCL: Primary cutaneous anaplastic large cell lymphoma; SPTCL: Subcutaneous panniculitis-like T-cell lymphoma; SS: Sézary syndrome.

|  | MF | SS | pcALCL | SPTCL |
| --- | --- | --- | --- | --- |
| Total Cases | 7,957 | 272 | 1126 | 205 |
| Total Deaths | 2,532 | 204 | 551 | 74 |
| Median follow up (range) (mo) | 66 (0 - 227) | 26 (0 -216) | 66 (0-226) | 48 (0 - 218) |
| SMR (95% CR) | 1.57 (1.49 - 1.65)* | 5.61 (4.65 - 6.7)* | 1.66 (1.48 - 1.86)* | 4.45 (3.26 - 5.93)* |
| AER per 1,000 population | 12.00 | 146.51 | 15.06 | 37.05 |
| Causes of death (no, %) |  |  |  |  |
| Cancer | 1416 (55.9) | 148 (72.5) | 305 (55.4) | 48 (64.9) |
| CTCL | 957 (37.8) | 129 (63.2) | 208 (37.7) | 37 (50.0) |
| HL | 7 (0.3) | 0 (0.0) | 2 (0.4) | 0 (0.0) |
| Non-lymphoma cancer | 452 (17.9) | 19 (9.3) | 95 (17.2) | 11 (14.9) |
| Non-cancer | 650 (44.1) | 56 (27.5) | 246 (44.6) | 26 (35.1) |
| Infection | 87 (3.4) | 11 (5.4) | 20 (3.6) | 4 (5.4) |
| Cardiovascular | 444 (17.5) | 23 (11.3) | 95 (17.2) | 10 (13.5) |
| Diabetes | 35 (1.4) | 0 (0.0) | 8 (1.5) | 0 (0.0) |
| Alzheimer's | 54 (2.1) | 1 (0.5) | 15 (2.7) | 0 (0.0) |
| COPD | 73 (2.9) | 3 (1.5) | 17 (3.1) | 0 (0.0) |
| Accident, homicide, etc | 38 (1.5) | 1 (0.5) | 12 (2.2) | 2 (2.7) |
| Other | 351 (13.9) | 16 (7.8) | 75 (13.6) | 8 (10.8) |
| Unknown | 34 (1.3) | 1 (0.5) | 4 (0.7) | 1 (1.4) |

**Table S3.** Causes of death by CTCL subtype

AER: Absolute excess risk; COPD: Chronic obstructive pulmonary disease; CTCL: Cutaneous T-cell lymphoma; HL: Hodgkin lymphoma; MF: Mycosis fungoides; pcALCL: Primary cutaneous anaplastic large cell lymphoma; SMR: standardized mortality ratio; SPTCL: Subcutaneous panniculitis-like T-cell lymphoma; SS: Sézary syndrome.

AER is per 1,000 persons per year.

* p < 0.05, relative to matched population controls

|  | MF | | SS | |
| --- | --- | --- | --- | --- |
|  | SMR | AER | SMR | AER |
| All causes | 1.57 (1.49 - 1.65)* | 11.998 | 5.61 (4.65 - 6.7)* | 146.514 |
| All malignant cancers | 3.66 (3.41 - 3.92)* | 13.252 | 17.52 (14.03 - 21.61)* | 121.903 |
| CTCL | 73.63 (67.84 - 79.79)* | 13.154 | 415.63 (329.57 - 517.29)* | 118.595 |
| Hodgkin lymphoma | 8.20 (1.69 - 23.96)* | 0.059 | 0 (0 - 466.85) | -0.012 |
| Skin cancer excluding basal and squamous | 2.96 (1.62 - 4.97)* | 0.207 | 0 (0 - 33.65) | -0.163 |
| Brain and CNS cancer | 2.87 (1.57 - 4.82)* | 0.204 | 9.87 (0.25 - 54.98) | 1.335 |
| Leukemia | 1.80 (1.03 - 2.93)* | 0.159 | 4.7 (0.12 - 26.16) | 1.17 |
| Other infection | 1.77 (1.01 - 2.87)* | 0.155 | 0 (0 - 22.0) | -0.249 |
| Septicemia | 1.44 (0.88 - 2.22) | 0.137 | 12.77 (3.48 - 32.69)* | 5.479 |
| Pneumonia and influenza | 1.12 (0.71 - 1.66) | 0.056 | 7.87 (2.14 - 20.14)* | 5.188 |
| Diseases of heart | 0.88 (0.77 - 1.01) | -0.655 | 1.76 (0.84 - 3.24) | 6.424 |

|  | pcALCL | | SPTCL | |
| --- | --- | --- | --- | --- |
|  | SMR | AER | SMR | AER |
| All causes | 1.66 (1.48 - 1.86)* | 15.061 | 4.45 (3.26 - 5.93)* | 37.048 |
| All malignant cancers | 4.00 (3.41 - 4.67)* | 15.562 | 12.55 (8.69 - 17.53)* | 32.514 |
| Non-Hodgkin Lymphoma | 77.54 (64.19 - 92.86)* | 14.919 | 341.98 (230.73 - 488.19)* | 31.082 |
| Hodgkin lymphoma | 0 (0 - 54.97) | -0.009 | 0 (0 - 794.68) | -0.005 |
| Skin cancer excluding basal and squamous | 1.09 (0.03 - 6.06) | 0.01 | 19.71 (0.5 - 109.79) | 0.986 |
| Brain and CNS cancer | 2.26 (0.27 - 8.15) | 0.143 | 0 (0 - 60.56) | -0.063 |
| Leukemia | 4.16 (1.67 - 8.57)* | 0.681 | 29.74 (6.13 - 86.91)* | 3.012 |
| Other infection | 2.62 (0.71 - 6.71) | 0.317 | 8.17 (0.21 - 45.53) | 0.912 |
| Septicemia | 0.78 (0.09 - 2.81) | -0.073 | 0 (0 - 21.6) | -0.177 |
| Pneumonia and influenza | 0.48 (0.06 - 1.72) | -0.281 | 9.80 (1.19 - 35.42)* | 1.866 |
| Diseases of heart | 0.86 (0.62 - 1.17) | -0.827 | 1.61 (0.44 - 4.12) | 1.573 |

**Table S4.** Standardized mortality ratio (SMR) and absolute excess risk (AER) by CTCL subtype

CTCL: Cutaneous T-cell lymphoma; HL: Hodgkin lymphoma; MF: Mycosis fungoides; pcALCL: Primary cutaneous anaplastic large cell lymphoma; SPTCL: Subcutaneous panniculitis-like T-cell lymphoma; SS: Sézary syndrome.

AER is per 1,000 persons per year.

* p < 0.05, relative to matched population controls

| MF | | | | | |
| --- | --- | --- | --- | --- | --- |
|  | All-cause mortality | All malignant tumors | CTCL | Septicemia | Pneumonia and influenza |
| All patients | 1.6 (1.5- 1.7)* | 3.7 (3.4- 3.9)* | 73.6 (67.8 - 79.8)* | 1.4 (0.9 - 2.2) | 1.1 (0.7 - 1.7) |
| White | 1.4 (1.4 - 1.5)* | 3.3 (3.1- 3.6)* | 60.9 (55.3 -66.9)* | 1.2 (0.6 - 2.0) | 1.0 (0.6 - 1.6) |
| Black | 2.5 (2.2 - 2.8)* | 5.9 (5.0 - 6.9)* | 241.1 (202.0 - 285.6)* | 3.0 (1.2 - 6.1)* | 1.5 (0.3 - 4.4) |
| SS | | | | | |
|  | All-cause mortality | All malignant tumors | CTCL | Septicemia | Pneumonia and influenza |
| All patients | 5.6 (4.7- 6.7)* | 17.5 (14.0 - 21.6)* | 415.6 (329.6 - 517.3)* | 12.8 (3.5 - 32.7)* | 7.9 (2.1 - 20.1)* |
| White | 4.8 (3.9 - 5.8)* | 14.8 (11.5 - 18.9)* | 328.9 (251.0 - 423.4)* | 14. (3.9 - 36.9)* | 4.2 (0.5 - 15.1) |
| Black | 13.6 (8.4 - 20.7)* | 38.9 (22.2 - 63.2)* | 1,854.2 (1,059.8 - 3,011.1)* | 0.0 (0 - 109.7) | 37.0 (0.9 - 206.4) |
| pcALCL | | | | | |
|  | All-cause mortality | All malignant tumors | CTCL | Septicemia | Pneumonia and influenza |
| All patients | 1.7 (1.5 – 1.9) | 4.0 (3.4 – 4.7) | 77.5 (64.2 – 92.9) | 0.8 (0.1 – 2.8) | 0.5 (0.1 – 1.7) |
| White | 1.6 (1.4 – 1.8) | 3.8 (3.1 – 4.4) | 68.7 (55.7 – 83.8) | 0.5 (0.0 – 2.5) | 0.5 (0.1 – 1.9) |
| Black | 2.9 (2.0 – 4.0) | 7.2 (4.5 – 10.8) | 294.9 (177.5 – 460.5) | 0.0 (0.0 – 13.0) | 0.0 (0.0 – 15.7) |
| SPTCL |  |  |  |  |  |
|  | All-cause mortality | All malignant tumors | CTCL | Septicemia | Pneumonia and influenza |
| All patients | 4.5* (3.3 – 5.9) | 12.6* (8.7 – 17.5) | 342.0* (230.7 – 488.2) | 0.0 (0.0 – 21.6) | 9.8* (1.2 – 35.4 |
| White | 4.6* (3.2 – 6.4) | 13.0* (8.5 – 19.1) | 300.3* (188.2 – 454.7) | 0.0 (0.0 – 33.7) | 12.7* (1.5 – 45.8) |
| Black | 3.5* (1.6 – 6.6) | 10.3* (4.2 – 21.3) | 518.9* (208.6 – 1069.0) | 0.0 (0.0 – 61.8) | 0.0 (0.0 – 83.1) |

**Table S5.** Standardized mortality ratio (SMR) by ethnicity and CTCL subtype

CTCL: Cutaneous T-cell lymphoma; MF: Mycosis fungoides; pcALCL: Primary cutaneous anaplastic large cell lymphoma; SPTCL: Subcutaneous panniculitis-like T-cell lymphoma; SS: Sézary syndrome.

* p < 0.05, relative to matched population controls

| MF | | | | | |
| --- | --- | --- | --- | --- | --- |
|  | 2-11 mo | 12-59 mo | 60-119 mo | 120+ mo | Total |
| Total Deaths, n (%) | 184 (100) | 660 (100) | 398 (100) | 232 (100) | 1,474 (100) |
| CTCL | 113 (61.4) | 320 (48.5) | 122 (30.7) | 41 (17.7) | 596 (40.4) |
| Non-lymphoma cancer | 11 (6.0) | 82 (12.4) | 79 (19.8) | 53 (22.8) | 225 (15.3) |
| Non-cancer | 60 (32.6) | 255 (38.6) | 197 (49.5) | 138 (59.5) | 650 (44.1) |
| SS | | | | | |
|  | 2-11 mo | 12-59 mo | 60-119 mo | 120+ mo | Total |
| Total Deaths, n (%) | 30 (100) | 69 (100) | 16 (100) | 5 (100) | 120 (100) |
| CTCL | 22 (73.3) | 49 (71.0) | 8 (50) | 1 (20) | 80 (66.7) |
| Non-lymphoma cancer | 1 (3.3) | 5 (7.2) | 0 (0) | 1 (20) | 7 (5.8) |
| Non-cancer | 7 (23.3) | 15 (21.7) | 8 (50) | 3 (60) | 33 (27.5) |
| pcALCL | | | | | |
|  | 2-11 mo | 12-59 mo | 60-119 mo | 120+ mo | Total |
| Total Deaths, n (%) | 59 (100) | 129 (100) | 80 (100) | 27 (100) | 295 (100) |
| CTCL | 40 (67.8) | 57 (44.2) | 18 (22.5) | 3 (11.1) | 118 (40) |
| Non-lymphoma cancer | 3 (5.08) | 23 (17.8) | 15 (18.8) | 5 (18.5) | 46 (15.6) |
| Non-cancer | 16 (27.1) | 49 (38.0) | 47 (58.8) | 19 (70.4) | 131 (44.4) |
| SPTCL | | | | | |
|  | 2-11 mo | 12-59 mo | 60-119 mo | 120+ mo | Total |
| Total Deaths, n (%) | 18 (100) | 18 (100) | 5 (100) | 5 (100) | 46 (100) |
| CTCL | 15 (83.3) | 11 (61.1) | 2 (40.0) | 2 (40.0) | 30 (65.2) |
| Non-lymphoma cancer | 0 (0) | 3 (16.7) | 1 (20.0) | 0 (0) | 4 (8.7) |
| Non-cancer | 3 (16.7) | 4 (22.2) | 2 (40.0) | 3 (60.0) | 12 (26.1) |

**Table S6.** Causes of Death by Latency

CTCL: Cutaneous T-cell lymphoma; MF: Mycosis fungoides; NR: Not reached; OS: Overall survival; pcALCL: Primary cutaneous anaplastic large cell lymphoma; SPTCL: Subcutaneous panniculitis-like T-cell lymphoma; SS: Sézary syndrome.

|  | Early Stage | Advanced Stage | Unknown Stage |
| --- | --- | --- | --- |
| MF | | | |
| Total Cases | 3086 | 655 | 5294 |
| Total Deaths | 838 | 379 | 1315 |
| SMR |  |  |  |
| All causes | 1.2 (1.1 – 1.3)* | 3.5 (3.1 – 3.9)* | 1.4 (1.3 – 1.4)* |
| CTCL | 49.5 (42.9 – 56.8)* | 270.4 (231.1 – 314.5)* | 57.7 (51.6 – 64.2)* |
| Causes of death, n (%) |  |  |  |
| CTCL | 269 (32.1) | 229 (60.4) | 459 (34.9) |
| Non-lymphoma cancer | 167 (19.9) | 43 (11.3) | 251 (19.1) |
| Non-cancer | 390 (46.5) | 104 (27.4) | 586 (44.6) |
| Unknown | 12 (1.4) | 3 (0.8) | 19 (1.4) |
| SS | | | |
| Total Cases | 13 | 125 | 192 |
| Total Deaths | 11 | 97 | 96 |
| SMR |  |  |  |
| All causes | 5.0 (2.1 – 9.8)* | 5.2 (4.0 – 6.5)* | 4.4 (3.4 – 5.6)* |
| CTCL | 407.1 (132.2 – 950.0)* | 399.1 (296.2 – 526.2)* | 355.1 (257.0 – 478.3)* |
| Causes of death, n (%) |  |  |  |
| CTCL | 7 (63.4) | 64 (66.0) | 58 (60.4) |
| Non-lymphoma cancer | 1 (9.1) | 8 (8.2) | 10 (10.4) |
| Non-cancer | 3 (27.3) | 24 (24.7) | 28 (29.2) |
| Unknown | 0 (0.0) | 1 (1.0) | 0 (0.0) |
| pcALCL | | | |
| Total Cases | 662 | 133 | 786 |
| Total Deaths | 218 | 85 | 239 |
| SMR |  |  |  |
| All causes | 1.3 (1.1 – 1.5)* | 2.9 (2.2 – 3.7)* | 1.5 (1.3 – 1.7)* |
| CTCL | 49.0 (35.9 – 65.3)* | 225.2 (155.9 – 314.7)* | 63.4 (48.0 – 82.1)* |
| Causes of death, n (%) |  |  |  |
| CTCL | 70 (32.1) | 52 (61.2) | 83 (34.7) |
| Non-lymphoma cancer | 41 (18.8) | 15 (17.6) | 41 (17.2) |
| Non-cancer | 104 (47.7) | 18 (21.2) | 114 (47.7) |
| Unknown | 3 (1.4) | 0 (0.0 | 1 (0.4) |
| SPTCL | | | |
| Total Cases | 61 | 62 | 113 |
| Total Deaths | 17 | 27 |  |
| SMR |  |  |  |
| All causes | 3.1 (1.6 – 5.4)* | 5.6 (3.4 – 8.7)* | 3.7 (2.4 – 5.5)* |
| CTCL | 275.2 (118.8 – 542.2)* | 362.2 (180.8 – 648.1)* | 242.0 (132.3 – 406.0)* |
| Causes of death, n (%) |  |  |  |
| CTCL | 10 (58.8) | 13 (48.1) | 14 (46.7) |
| Non-lymphoma cancer | 1(5.9) | 6 (22.2) | 4 (13.3) |
| Non-cancer | 6 (35.3) | 7 (25.9) | 12 (40.0) |
| Unknown | 0 (0.0) | 1 (3.7) | 0 (0.0) |

**Table S7.** Causes of death by stage and CTCL subtype

CTCL: Cutaneous T-cell lymphoma; MF: Mycosis fungoides; pcALCL: Primary cutaneous anaplastic large cell lymphoma; SMR: Standardized mortality ratio; SPTCL: Subcutaneous panniculitis-like T-cell lymphoma; SS: Sézary syndrome.

* p < 0.05, relative to matched population controls
